# Decreased expression of microRNA-126 is associated with poor prognosis in patients with cervical cancer

**DOI:** 10.1186/s13000-014-0220-x

**Published:** 2014-12-31

**Authors:** Yao Yang, Kun-ling Song, Hong Chang, Long Chen

**Affiliations:** Department of Gynecology, Qingdao Municipal Hospital, No.1 Jiaozhou Road, Qingdao, 266011 People’s Republic of China; Department of Gynecology, People’s hospital of Sanya, Sanya, 572000 China; Department of Pathology, Qingdao Municipal Hospital, Qingdao, 266011 China

**Keywords:** miRNA, miR-126, Cervical cancer, Quantitative RT-PCR, Prognosis

## Abstract

**Background:**

MicroRNA-126(miR-126) has been shown to be frequently down-regulated in a variety of malignancies and act as a potential tumor suppressor. However, its correlations with the clinicopathological characters of cervical cancer remain unclear.

**Methods:**

TaqMan quantitative RT-PCR was used to determine the expression level of miR-126 in tissue samples. The associations of miR-126 expression with clinicopathologic variables were analyzed. Kaplan-Meier survival analysis was performed to analyze the association of miR-126 expression with overall survival (OS) of patients. Univariate and multivariate Cox regression analyses were performed.

**Results:**

miR-126 expression level in human cervical cancer tissues was significantly lower than that in adjacent nontumorous tissues (mean ± SD: 0.59 ± 0.44 vs. 1.00 ± 0.51, *P* < 0.0001). Decreased miR-126 expression in cervical cancer was found to be significantly associated with lymphatic invasion (*P* = 0.002), distant metastasis (*P* < 0.001), FIGO stage (*P* = 0.009), and histological grade (*P* = 0.005). Kaplan-Meier analysis showed that patients with lower levels of miR-126 had significantly poorer survival than those with higher expression of this miRNA in patients, with a 5-year OS of 45.7% and 70.9%, respectively (*P* = 0.002). Multivariate analysis revealed that miR-126 expression (HR = 3.97, 95% CI: 2.01-20.22; *P* = 0.003) was independently associated with the OS.

**Conclusion:**

Our data suggests the potential of miR-126 as a prognostic biomarker for cervical cancer.

**Virtual Slides:**

The virtual slide(s) for this article can be found here: http://www.diagnosticpathology.diagnomx.eu/vs/13000_2014_220

## Background

Cervical cancer is the second leading cause of death among women worldwide, with an estimated 530,000 deaths per year [1]. Although radiotherapy, chemotherapy and surgery have been recently used as standard treatment modalities for patients with cervical cancer, with consequent disease remission, clinical outcomes vary significantly between patients and can be difficult to predict. Therefore, it is important to understand the complete knowledge of the molecular biology, genetics, causes and cellular origin of cervical cancers which are of value in the development of improved therapeutic strategies and in the identification of prognostic markers [2].

MicroRNAs (miRNAs) are small, conserved, non-coding short RNAs of 18–25 nucleotides in length that bind to target mRNAs mainly at their 3’-untranslated region (UTR) [3]. Many miRNAs have been implicated as key regulators of cellular growth and differentiation and have been found to deregulate proliferation in human cancers [4-6]. In human cancers, the expression of miRNAs is generally down-regulated or up-regulated in malignant tissues compared with the corresponding nonmalignant tissues, suggesting the deregulation of miRNA expression and the contribution of miRNAs to the multistep processes of carcinogenesis, either as oncogenes or as tumor-suppressor genes [7-9].

MiR-126 is frequently down-regulated in a variety of malignancies and acts as a potential tumor suppressor [10-12]. Moreover, low expression of miR-126 has been found to be correlated with poor prognosis in patients with breast cancer, adult T cell leukemia, colorectal cancer(CRC) and malignant mesothelioma [13-15]. Previously, miR-126 expression level in cervical cancer tissues was found to be significantly decreased compared with that in normal cervical tissues (*P* <0.01). However, the clinical significance and prognostic value of miR-126 in cervical cancer have not been investigated.

## Methods

### Patients and tissue samples

Fresh cervical cancer and matched adjacent normal tissue specimens were collected from 133 patients who underwent surgery between March 2008 and July 2013 in the Department of Gynecology, Qingdao Municipal Hospital. The corresponding adjacent normal tissues were obtained 3 cm beyond the boundary of cervical cancer tissues. The fresh tissue specimens were immediately frozen in liquid nitrogen until use. The selection criteria for patients with cervical cancer were as follows: (1) pathologically confirmed patients with cervical cancer; (2) the patients had no history of other cancers. No patients had preoperative chemotherapy, radiotherapy, or other treatment history or other inflammatory diseases. Patient’s conditions were staged according to the criteria of the International Federation of Gynecology and Obstetrics (FIGO). The clinicopathologic features of all the patients were summarized in Table 1. Overall survival(OS) time was calculated from the date of the initial surgical operation to death. Follow-up information of all patients was updated every 3 months for the first 2 years, every 4 months for the third year, every 6 months for the fourth and fifth years, and then every year thereafter by telephone visit and questionnaire letters. Death of the participants was ascertained by reporting from the family and verified by review of public records.

**Table 1:** **The relationship between miR-126 expression and clinicopathological characteristics in 133 patients with cervical cancer**

**Parameters**	**Number of cases**	**miR-126 expression**		
		**Low**	**High**	**P value**
Age (y)				
<65	56	29	27	0.46
≥ 65	77	33	44	
HPV				
(+)	97	47	50	0.21
(−)	36	15	21	
Tumor histology				
Squamous	101	54	47	0.19
Adenocarcinoma	22	5	17	
Clear cell	10	3	7	
Tumor Size (cm)				
<4	54	19	35	0.07
≥4	79	43	36	
Lymphatic invasion				
Yes	63	45	18	0.002
No	70	17	53	
Distant metastasis				
Yes	28	23	5	<0.001
No	105	39	66	
FIGO stage				
I/II	62	20	42	0.009
III/IV	71	42	29	
Histological grade				
Well/moderate	82	25	57	0.005
Poor	51	37	14	

This study was approved by the Research Ethics Committee of the Qingdao Municipal Hospital. Written informed consent was obtained from all of the patients.

All specimens were handled and made anonymous according to the ethical and legal standards.

### RNA extraction and Real-Time RT-PCR

Total RNA was extracted with Trizol reagent (Invitrogen, Carlsbad, CA, USA) according to the manufacturer’s instructions. The concentration and purity of all RNA samples were detected by NanoDrop ND-2000 spectrophotometer (NanoDrop Technologies, Houston, TX, USA). NCode™ SYBR® Green miRNA qRT-PCR Kit (Invitrogen, Carlsbad, CA, USA) was used to synthesize specific cDNA of miR-126 and U6B(as an internal control), and perform qRT-PCR, which was analyzed with the DNA Engine Opticon 2 Real-Time Cycler (MJ Research Inc., Waltham, MA, USA) according to the manufacturer’s instructions. Each sample was examined in triplicate and analyzed by the comparative threshold cycle (Ct) method. The expression levels of miR-126 were normalized to U6B.

### Statistical analysis

Statistical analysis was conducted using the SPSS 18.0 for Windows (SPSS Inc., Chicago, IL, USA). The chi-square test was used to assess miR-126 expression with respect to clinicopathological parameters. The survival curves of the patients were determined using the Kaplan-Meier method and Cox regression, and the log-rank test was used for statistical evaluations. Univariate Cox regression was performed on each clinical covariate to examine its influence on patient survival. Final multivariate models were based on step-wise addition. A Wald statistic of *P* < 0.05 was used as the criterion for inclusion in final multivariate models. Data were expressed as the mean and standard deviation and analyzed using one-way analysis of variance. *P* < 0.05 was considered to indicate a significant difference.

## Results

### The expression level of miR-126 in cervical cancer

**Figure 1:**
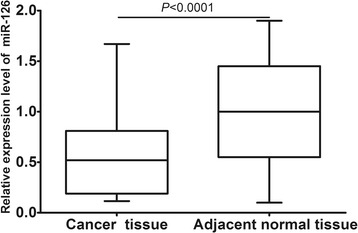
**miR-126 expression levels in cervical cancer tissues and adjacent non-tumorous tissues.** miR-126 expression in cervical cancer tissues was significantly lower than that in adjacent nontumorous tissues (mean ± SD: 0.59 ± 0.44 vs. 1.00 ± 0.51, P < 0.0001).

qRT-PCR was used to assess the expression of miR-126 in cervical cancer tissues and adjacent non-tumorous tissues. The results showed that miR-126 expression level in human cervical cancer tissues was significantly lower than that in adjacent nontumorous tissues (mean ± SD: 0.59 ± 0.44 vs. 1.00 ± 0.51, *P* < 0.0001; shown in Figure 1).

### Correlation of miR-126 expression with clinicopathological characteristics

The Median expression level of miR-126 was used as a cut-off point to divide all 133 patients into two groups: cervical cancer patients who expressed miR-126 at levels less than the cut-off value were assigned to the low expression group (n =62), and those with expression above the cut-off value were assigned to the high expression group (n =71). The relationships between miR-126 expression levels and different clinicopathological factors were shown in Table 1. Decreased miR-126 expression in cervical cancer was found to be significantly associated with lymphatic invasion (*P* = 0.002), distant metastasis (*P* < 0.001), FIGO stage (*P* = 0.009), and histological grade (*P* = 0.005). However, no significant correlation was observed between miR-126 expression and other clinicopathologic variables, such as age, HPV infection, tumor histology, and tumor size (all *P* > 0.05).

### Relationship between miR-126 expression and cervical cancer patients’ survival

**Figure 2:**
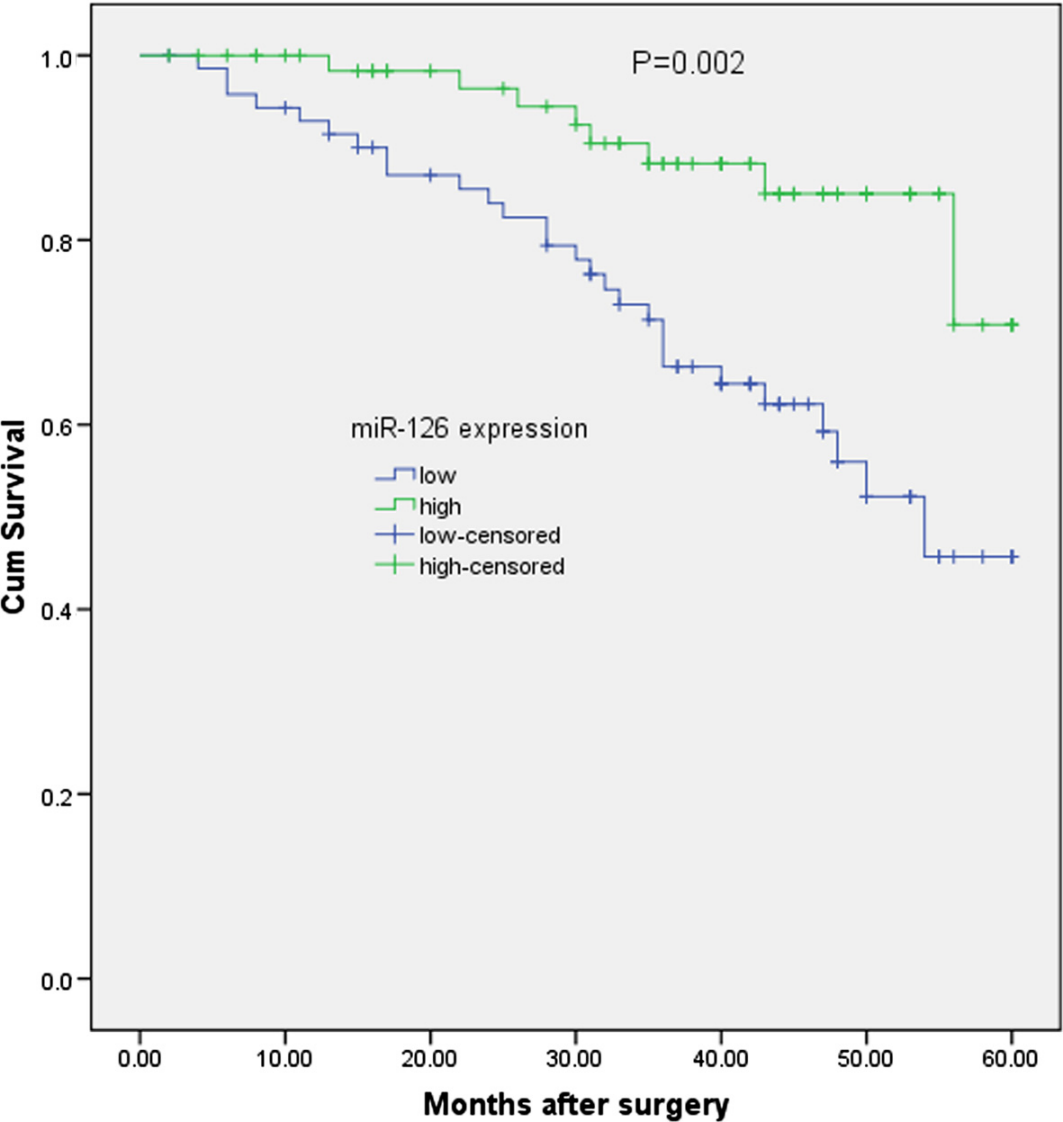
**Survival analysis of the miR-126 expression levels with overall survival of patients with cervical cancer after surgery.**

To evaluate whether miR-126 expression can predict cervical cancer prognosis, we next performed survival analysis. Kaplan-Meier analysis showed that patients with lower levels of miR-126 had significantly poorer survival than those with higher expression of this miRNA in patients, with a 5-year OS of 45.7% and 70.9%, respectively(*P* = 0.002; shown in Figure 2).

Univariate and multivariate analyses were utilized to evaluate whether the miR-126 expression level and various clinicopathological features were independent prognostic parameters of patient outcomes. Multivariate analysis revealed that miR-126 expression (HR = 3.97, 95% CI: 2.01-20.22; *P* = 0.003), distant metastasis (HR = 4.78, 95% CI: 2.31-20.12; *P* = 0.004), and FIGO stage (HR = 2.12, 95% CI: 1.68-18.29; *P* = 0.001) were independently associated with the overall survival (shown in Table 2).

**Table 2:** **Multivariate Cox’s hazards model analysis for prognostic factors**

**Variable**	**Hazard ratio**	**95% CI**	**P value**
Age (y)	1.02	0.29-2.88	0.66
HPV	1.28	0.79-2.44	0.11
Tumor Size (cm)	2.11	0.81-3.19	0.09
Tumor histology	0.91	0.25-1.91	0.81
Lymphatic invasion	3.14	0.88-19.23	0.06
Distant metastasis	4.78	2.31-20.12	0.004
FIGO stage	2.12	1.68-18.29	0.001
Histological grade	5.11	0.91-17.89	0.07
miR-126 expression	3.97	2.01-20.22	0.003

## Discussion

Accurate prediction of the prognosis for the individual patient with cervical cancer is of great importance, and molecular biomarkers that could be served as prognostic markers would be useful in determining an individualized treatment plan for a cervical cancer patient. However, the biomarkers used in this tumor group today are not satisfactory, and it is needed to exploit additional markers to fine-tune this process.

MiRNAs, a class of naturally occurring, non-coding, short single stranded RNAs, are deregulated in cancer, and they are involved in malignant transformation and tumor development. In recent years, numerous studies have shown aberrant expression of miRNAs in human cancers, including cervical cancer, some of which function as tumor suppressor genes or oncogenes. Due to their tissue-and disease-specific expression patterns and tremendous regulatory potential, miRNAs are being identified as diagnostic and prognostic biomarkers, as well as additional therapeutic tools [16,17]. As more and more studies report the relationships between miRNAs and cervical cancer, its potential as novel biomarkers in cervical cancer is growing [18-20].

MiR-126, derived from a common precursor structure located within the epidermal growth factor-like domain 7 (EGFL7) gene, is frequently down-regulated in a variety of malignancies and acts as a potential tumor suppressor [10-12]. Previous studies have reported that miR-126 may play a role in tumorigenesis and growth by regulating the vascular endothelial growth factor (VEGF)/phosphoinositol 3-kinase (PI3K)/AKT signaling pathways in human breast cancer [21]. Additionally, this miRNA may function as a tumor suppressor, with Crk as a direct target, in gastric cancer [22] and via the regulation of ADAM9b in pancreatic cancer [23]. miR-126 may also play a role in angiogenesis in ischemia [24], and has also been reported to enhance the sensitivity of non-small cell lung cancer(NSCLC) cells to anticancer agents by targeting VEGF-A [25]. Together, these previous studies have demonstrated the important role of miR-126 in various cancers. Moreover, low expression of miR-126 has been found to be correlated with poor prognosis in patients with breast cancer, adult T cell leukemia, CRC and malignant mesothelioma [13-15]. Previously, miR-126 expression in cervical cancer tissues was found to be significantly decreased compared with that in normal cervical tissues. Yu et al. found that miR-126 was able to suppress the proliferation of cervical cancer cells and alters cell sensitivity to the chemotherapeutic drug bleomycin [26]. However, the clinical significance and prognostic value of miR-126 in cervical cancer have not been investigated. In the present study, we showed that miR-126 was significantly down-regulated in cervical cancer tissues for the first time. Decreased miR-126 expression in cervical cancer was found to be significantly associated with lymphatic invasion, distant metastasis, FIGO stage, and histological grade, suggesting that miR-126 might be involved in the carcinogenesis and metastasis of cervical cancer. More importantly, we proved that patients with a lower expression of miR-126 tended to have shorter survival than patients with higher levels. Furthermore, multivariate analysis revealed that miR-126 expression was independently associated with the OS, indicating that lower miR-126 level was a marker of poor prognosis for patients with cervical cancer.

## Conclusions

In conclusion, this study indicated that down-regulation miR-126 was associated with tumor progression and poor prognosis in cervical cancer and was identified for the first time as an independent poor prognostic factor for patients with cervical cancer. Further study with a larger case population is needed to confirm the prognostic value of miR126 expression in cervical cancer.
